# Management of Chronic Myeloid Leukemia in Children and Young Adults

**DOI:** 10.1007/s11899-022-00673-5

**Published:** 2022-08-03

**Authors:** Maegan Ford, Michael Mauro, Catherine Aftandilian, Kathleen M. Sakamoto, Nobuko Hijiya

**Affiliations:** 1 Division of Pediatric Hematology/Oncology/Stem Cell Transplant, Department of Pediatrics, Columbia University Irving Medical Center, New York, NY 10069 USA; 2 Myeloproliferative Neoplasms Program, Memorial Sloan Kettering Cancer Center, New York, NY 10065 USA; 3 Division of Hematology/Oncology, Department of Pediatrics, Stanford University, Stanford, CA 94305 USA; 4 Division of Hematology, Oncology, Stem Cell Transplant & Regenerative Medicine, Department of Pediatrics, Stanford University, Stanford, CA 94304 USA

**Keywords:** Chronic myeloid leukemia, CML, Tyrosine kinase inhibitors, TKI, BCR-ABL1, Children, Young adults

## Abstract

**Purpose of Review:**

Due to lack of pediatric-specific data, the management of chronic myeloid leukemia (CML) in pediatric, adolescents, and young adults is guided by adult CML evidence-based recommendations. Pediatric CML presents differently than adult CML and is often a more aggressive disease with different biological and host factors, yet there is sparse literature on how to address those differences.

**Recent Findings:**

Over the past two decades, tyrosine kinase inhibitors (TKIs) have changed the way CML is treated. There are currently three FDA-approved TKIs (imatinib, dasatinib, and nilotinib) for pediatric patients. When choosing which TKI to begin treatment with, there are many factors that should be considered on a case-to-case basis to obtain optimal outcomes. The safety profiles for long-term TKI use in pediatrics require further study. Unlike adults, children are still actively growing during TKI use, and the effect on development can be detrimental. TKI therapy is not recommended during pregnancy with variable but significant risk of fetal abnormalities and miscarriage, warranting counseling for young female patients prior to beginning TKIs. Attempts for treatment-free remission (TFR) by planned TKI cessation in eligible adult patients in deep and sustained molecular remission are now done as a standard of practice. However, data is sparse in the pediatric population. There is currently an ongoing Children’s Oncology Group (COG) study to determine the feasibility of TFR as a treatment goal.

**Summary:**

Further research and additional pediatric trials are needed to characterize the unique aspects of CML in children and adolescents and optimize outcomes.

## Introduction

Chronic myeloid leukemia (CML) is a myeloproliferative neoplasm that results from translocation t(9:22) and the resultant BCR-ABL1 fusion. CML is rare in pediatrics, adolescents, and young adults. It comprises approximately 2–3% of all pediatric leukemias in those under 15 years old and approximately 9% of all leukemias in those between 15 and 19 years old [1, 2]. Given its lower incidence, most of the data used to guide management is derived from the adult population. However, there is no clear evidence that risk assessment tools and adult treatment guidelines can be applied to pediatric patients. There are data suggesting that CML is often more aggressive in pediatric patients for many different reasons, such as underlying biology and host factors, necessitating integration into optimal treatment of CML in younger individuals [3, 4].

Over the last two decades, tyrosine kinase inhibitors (TKIs) have revolutionized the way CML is treated, including the role of hematopoietic stem cell transplant (HSCT). HSCT is now typically reserved for those patients who cannot tolerate or are resistant to TKIs, or those who present or progress to blast crisis [2]. CML is now beyond simply a chronic disease, given the novel paradigm of long-term treatment with TKIs, often for decades to keep disease under control. Such a paradigm is of even greater importance to younger individuals.

Unlike adults, children are actively developing and growing while undergoing TKI therapy. Long-term side effects of treatment of chronic CML with TKI over decades in children and young adults are still unknown [5]. In adult patients, recommendations to consider when potentially discontinuing TKIs are included in The National Comprehensive Cancer Network (NCCN) Guidelines [6] and European Leukemia Net (ELN) guidelines [7]. Pediatric patients would benefit, potentially even more so, from discontinuing TKI therapy at the earliest point possible to limit the potential developmental side effects. However, there are sparse data supporting or guiding the timing of such an endeavor in younger patients [2]. This review will encompass the specific treatment and management considerations for children and young adults with CML, and how to best transition them to adult care.

## Consideration of Differences in Children and Young Adults

Pediatric patients with CML present differently than adults, usually manifesting with more aggressive disease features (Table 1); the biology of CML in children and adolescents thus should not be assumed to be the same as in adults [3]. Children and adolescents typically present with a higher white blood cell (WBC) count and more circulating blasts at diagnosis. Median WBC counts in adults at diagnosis range from 50 to 70 × 10^9^/L [8], but in children and adolescents, the initial WBC count is often as high as 300 × 10^9^/L [9]. Children and adolescents more often have significant splenomegaly and profound anemia, and unfortunately present in an advanced stage of CML — either in the accelerated or blast phase [4].

**Table 1:** Differences between pediatric and adult CML

	Pediatric CML	Adult CML
Presentation	Higher WBC count More frequently in advanced stage More significant splenomegaly More profound anemia	Lower WBC count More frequently in chronic phase
Genomics	Higher proportion of bcr-abl breakpoints within *Alu* repeat regions and telomeres Down regulation of genes in the Rho-GTPase pathway	Higher proportion of DNA fusion sites within centromeres
Treatment	No evidence-based treatment guidelines	Multiple prognostic scoring systems NCCN and ELN guidelines

Approximately 5–15% of both children and adults harbor additional cytogenetic abnormalities (ACAs) including variant translocations, additional chromosomal abnormalities, and complex karyotypes [10, 11]. There is data suggesting ACAs in children may not be associated with inferior outcomes unlike adults. From a genomic standpoint, pediatric patients also appear to have a more complex profile and may harbor a higher proportion of altered oncogenes. CML in children has different breakpoint patterns in the BCR gene, specifically a higher proportion within the Alu repeat regions. They show a distribution of BCR-ABL1 breakpoints that resembles Philadelphia chromosome positive acute lymphoblastic leukemia, which all may correlate with more aggressive disease [2, 3]. Several mutations have been implicated in CML across all ages, including ASXL1, in addition to deregulated genes in the PI3K/AKT/WNT/beta-catenin, Sonic Hedgehog, and MAPK pathways [12]. ASXL1 mutations may be more frequent in pediatric patients with CML compared with adults. One report found that 29% of pediatric and young adult patients with chronic phase CML had an ASXL1 mutation compared with 7–13% of adults [13]. Recent data demonstrated that CD34 + cells from children with chronic CML have different transcriptomes compared to adults. RNA-sequencing results showed that genes in the Rho-GTPase pathway were significantly downregulated in pediatric CML, but not adult CML patient CD34 + cells [14].

There are no standard pediatric specific guidelines in the care of pediatric and adolescent CML patients; thus, many pediatric oncologists are forced to rely heavily on recommendations for adult CML. Treatment guidelines (like NCCN and ELN) which guide therapy changes and define failure, based on cytogenetic and molecular responses to TKI therapy, have never been validated in children [4, 5]. The majority of prognostic scoring systems, like Sokal, EURO, EUTOS, and Hasford, which are used in adults, have not been validated in pediatric populations and thus may be unreliable in general. Interestingly, a study from the International Registry for Childhood Chronic Myeloid Leukemia recently evaluated 4 prognostic scoring systems (Sokal, Euro, EUTOS, and EUTOS Long-Term Survival) in 350 pediatric patients with newly diagnosed chronic phase CML treated with imatinib. The EUTOS Long-Term survival score showed better differentiation of progression-free survival than the other scoring systems. Future studies should examine whether children in the high-risk group might benefit from the use of second-generation TKIs as initial therapy [15].

It is important to note that children may require a lengthier course of TKI therapy, simply given a dramatically longer life expectancy, creating many obstacles in pediatric and adolescent patients. Adolescents and young adults are known to be less compliant with routine medication administration, which is not ideal when a medication may be used for decades. Longer use of TKIs, especially in cases of suboptimal response, may foster greater risk of resistance permitting disease progression to advanced stages of CML. Despite the advent of TFR, across all ages, the safety (and efficacy) of very long term TKI use has not been studied and is unknown. Immune dysfunction, thyroid, cardiac, liver, and fertility issues have all been reported in adults with CML, and particular focus is being paid to cardiovascular and vascular risk over time; there are no long-term data for adverse effects of TKI therapy in children [3, 4].

## Choosing Initial Treatment

TKIs have become the standard of care in CML patients in the chronic phase (CP); therefore, HSCT is no longer the definitive therapy for cure. There are three FDA-approved TKIs for first-line CML therapy use in pediatrics — imatinib (approved in 2003), dasatinib (approved in 2017), and nilotinib (approved in 2018). Bosutinib and ponatinib are both currently approved for adults, and the novel “STAMP” (allosteric inhibitor targeting the myristoyl pocket of ABL1) asciminib was approved for adults by the FDA in 2021 [16, 17]. There are open clinical trials for use in children [5, 18] for these three drugs (NCT04258943, NCT03934372, NCT04925479, respectively).

The TKI chosen for treatment should be based upon the aggregate of multiple factors leading to the best compliance and outcome (Table 2). Imatinib and dasatinib are given once a day with or without food, while nilotinib is given twice a day without food 2 h prior and 1 h after administration, potentially difficult for a young child or adolescent. Imatinib has generic formulations available, lending financial desirability to the health care system and theoretically for patients, given the potential of multiple decades of treatment for young patients. The second-generation TKIs (dasatinib and nilotinib) have been shown to induce a faster and deeper molecular response in adult patients in randomized trials [19, 20]. Although there are no randomized trials in pediatrics due to the small number of patients, some published studies show similar trends [2, 5, 21–23]. Although a faster and deeper molecular response has not been shown to have a survival benefit, it may be beneficial by accelerating the timeframe and number of eligible patients for attempt at discontinuation of TKI therapy to achieve TFR [2]. There is not currently enough data to use ACAs or prognostic scoring systems to guide initial treatment in pediatric patients. Given the faster and deeper response with second generation TKIs in adults, some providers may elect to start with a second generation TKI because there are few serious side effects such as cardiovascular events in pediatric patients, if they anticipate it will be affordable.

**Table 2:** Comparison of TKIs

	FDA approved in pediatrics?	Generation	Dosing frequency	Unique toxicities	ABL mutation associated with resistance	Other
Imatinib	Yes	First	Once daily With or without food	Muscle cramps Edema Diarrhea	Too many to list	
Dasatinib	Yes	Second	Once daily With or without food	Pleural/pericardial effusions Pulmonary hypertension GI bleeding QTc prolongation	T315I/A, F317L/V/I/C, V299L	Crosses the blood brain barrier
Nilotinib	Yes	Second	Twice daily No food 2 h prior and 1 h after	QTc prolongation Arterial occlusion Metabolic changes (glucose/lipids)	T315I, Y253H, E255K/V, F359V/C/I	
Bosutinib	No	Second	Daily With food	Diarrhea Hepatic enzyme increase	T315I, V299L, G250E, F317L	
Ponatinib	No	Third	Daily With or without food	Arterial and venous thrombosis Pancreatitis	Rare	Effective with T315I mutation
Asciminib	No	Third	Once or twice daily, without food	Myelosuppression Pancreas enzyme elevation, pancreatitis Hypertension	Rare	Effective with T315I mutation

Although TKIs are well tolerated in general, severe side effects associated with second- and third-generation TKIs have been reported in adult patients (Table 2). Cardiovascular side effects, specifically vaso-occlusive events, are of particular concern with the use of second- and third-generation TKIs in adults [24]. While this can cause provider reluctance to use second- and third-generation TKIs especially in older adults with preexisting morbidities [25], there are no serious side effects published within the pediatric literature to date [21, 22]. In addition, comorbidities in children are less common; therefore, there may be less concern for these side effects with later generation TKIs. In conclusion, many factors, including efficacy, cost, availability, toxicity profiles, and comorbidities, must be taken into account when choosing the best TKI for a specific pediatric patient with newly diagnosed CML in CP [2, 5].

There are no current TKI response criteria available to guide the specific management of pediatric patients. Pediatric providers thus resort to using the adult recommendations, such as the NCCN guidelines or ELN criteria [2]. These response criteria recommendations are based on adult data but likely correlate equally to disease modification and risk reduction and may reasonably be used for pediatric patients [1].

## Management of Refractory and Intolerant Disease

The most common cause of refractory disease in pediatric CML patients is noncompliance, especially in the adolescent and young adult age groups; the same is often cited in adults. Noncompliance/nonadherence often occurs early, often after 1 year of treatment given good response and patients feeling less burdened by their disease [1]. It is important to emphasize compliance at every visit with not only the patient, but also the caregivers, parents, or others. If a patient is not responding well to TKI therapy, noncompliance should be suspected and queried before investigating for resistance and switching TKIs [2]. TKI plasma trough levels are not routinely measured for compliance. If noncompliance is suspected but not confirmed by the patient, it may be possible to send a TKI plasma trough level for some TKIs to ensure an adequate drug level [1]. Lastly, review of concomitant medications, in addition to supplements, nontraditional medications, over-the-counter products, and dietary habits, is important to rule out drug-drug interactions, many of which may reduce patient exposure to their CML medication.

It is also important to review the potential and actual barriers for compliance with each patient to facilitate solutions. It is imperative to provide and review adherence tools, such as using a pillbox, setting a phone alarm, or marking administration on a calendar; having a routine schedule will lead to better compliance. Potential side effects of TKIs should be reviewed at each visit. As an example, GI toxicity with imatinib may manifest as diarrhea; having this adverse event chronically, even if not higher grade or constant, may not seem “dire,” but may specifically impact a patient’s extracurricular activities, a strong impetus to stop/reduce medication. Financial burden of the medication should also be reviewed; in younger patients, this is often the responsibility of others, and all parties need to understand the importance of consistent treatment. Addressing all the potential obstacles in a transparent and facilitating manner will only provide for better compliance and outcome in the long-term [1, 2].

If noncompliance has been ruled out, and the patient is still not responding to TKI therapy, resistance should be a concern. BCR-ABL1 mutation analysis is indicated for resistance; NCCN guidelines recommend that mutational analysis be sent after initiating TKI therapy if there is a suboptimal response to therapy, failure of prior response, or escalation into advance stage CML [1]. Based on specific types of resistance, TKI choice may be tailored to optimize disease sensitivity (Table 2).

## Issues with Side Effects in Children and Young Adults

Two decades after the approval of the first generation TKI imatinib for children, the long-term side effects in children and young adults remain less well understood. Pediatric patients often begin TKIs before or during puberty, which can cause detrimental effects on growth and development, but the exact mechanism is unclear [21, 26–28]. TKIs not only inhibit BCR-ABL1, but other targets as well, including PDGFR-beta signaling, resulting in reduced osteoclast activity and recruitment of chondrocytes in the growth plate, which is one of the proposed mechanisms for growth delay. This adversely affects overall bone metabolism and linear growth [1]. Thyroid and gonadal dysfunction are also seen and should be routinely monitored [2]. Metabolic derangements, particularly diabetes mellitus, have also been documented with nilotinib use, so routine blood glucose levels should be checked [1].

All patients being treated with TKI therapy should be educated on reproductive health and safe sexual practices. TKIs are teratogenic and have been shown to cause fetal abnormalities or spontaneous abortions, particularly in the first trimester [6]. Females should be counseled on safe sexual practices to avoid pregnancy entirely while on TKI therapy [4]. There should be a prolonged washout period after TKI discontinuation before attempting to conceive and therapy should be held throughout the pregnancy. If treatment is needed during pregnancy, interferon therapy (interferon alfa a2 or peginterferon alfa a2) can be considered, especially in the second trimester of pregnancy and beyond [6, 29]. TKI therapy can be resumed immediately after pregnancy, but mothers are advised not to breastfeed as it has been shown that imatinib and likely other TKIs can be transmitted through breast milk [1]. There have been no reported adverse effects or congenital abnormalities in the offspring of males who were undergoing TKI therapy at the time of conception [30]. There is sparse literature on fertility and the ability to reproduce after a prolonged TKI treatment course [4].

## Treatment-Free Remission in Pediatric Patients

There are very limited data regarding the feasibility of discontinuing TKIs in the pediatric and adolescent population once deep and sustained molecular remission is achieved. There are strict NCCN guidelines for discontinuing TKIs in adults, which include: age ≥ 18 years old, in chronic phase CML with no history of advance stage CML, on TKI therapy for at least 3 years, stable molecular response (MR4 or better) for ≥ 2 years, access to reliable qPCR testing, and frequent molecular monitoring [6]. It is important to note that within 6 months of stopping TKI therapy, 50–60% of patients do not stay in treatment-free remission (TFR) and have disease recurrence. This can be reversed by reinitiating TKI therapy, with remarkably high rates of return to remission/deep remission; patients with unsuccessful TFR may then require longer/indefinite duration of therapy [5]. There also have been reported clinical side effects consistent with “withdrawal syndrome” upon stopping TKIs, such as musculoskeletal pain and neurocognitive deficits. Withdrawal side effects could be more severe in pediatric patients and require prospective trials for further investigation [2]. In pediatrics, there is an ongoing Children’s Oncology Group study to assess feasibility of TFR (NCT03817398). Given that data remains limited in pediatrics, we currently do not recommend routine discontinuation of TKIs outside of a clinical trial.

## Transitioning from Pediatric to Adult Care

Transitioning pediatric patients to adult CML care should be fluid and tailored as it may vary on a case-by-case basis depending on the needs of that individual at the time. Transition to an adult clinic may be considered when patient is 18 years of age but may take place by the time a patient is 21 years of age. This transition is unique given the long-term relationship between patient and provider teams and chronicity of active treatment. Like any medical transition, clear communication between the teams to ensure seamless care is paramount [1].

## Conclusions

Pediatric CML care is ultimately based upon adult guidelines. There are documented host and molecular differences in CML between pediatric and adult patients, but how those differences affect treatment and prognosis are poorly understood. While TKIs have completely changed the way CML is treated and improved overall survival, there is little known about the side effects of decadelong TKI use. Additional study and pediatric trials are needed to characterize the unique aspects of CML in children, adolescents, and young adults.

## Declarations

N/A
